# Learning strategy impacts medical diagnostic reasoning in early learners

**DOI:** 10.1186/s41235-023-00472-3

**Published:** 2023-03-09

**Authors:** Signy Sheldon, Carina Fan, Idil Uner, Meredith Young

**Affiliations:** 1 Department of Psychology, McGill University, 2001 McGill College Avenue, Montreal, QC H3A 1G1 Canada; 2 Department of Psychology, University of Toronto, Toronto, Canada; 3 Institute of Health Sciences Education, McGill University, Montreal, Canada

**Keywords:** Clinical reasoning, Case-based reasoning, Expert development, Transfer of learning, Rehearsal effects

## Abstract

Relating learned information to similar yet new scenarios, transfer of learning, is a key characteristic of expert reasoning in many fields including medicine. Psychological research indicates that transfer of learning is enhanced via active retrieval strategies. For diagnostic reasoning, this finding suggests that actively retrieving diagnostic information about patient cases could improve the ability to engage in transfer of learning to later diagnostic decisions. To test this hypothesis, we conducted an experiment in which two groups of undergraduate student participants learned symptom lists of simplified psychiatric diagnoses (e.g., Schizophrenia; Mania). Next, one group received written patient cases and actively retrieved the cases from memory and the other group read these written cases twice, engaging in a passive rehearsal learning strategy. Both groups then diagnosed test cases that had two equally valid diagnoses—one supported by “familiar” symptoms described in learned patient cases, and one by novel symptom descriptions. While all participants were more likely to assign higher diagnostic probability to those supported by the familiar symptoms, this effect was significantly larger for participants that engaged in active retrieval compared to passive rehearsal. There were also significant differences in performance across the given diagnoses, potentially due to differences in established knowledge of the disorders. To test this prediction, Experiment 2 compared performance on the described experiment between a participant group that received the standard diagnostic labels to a group that received fictional diagnostic labels, nonsense words designed to remove prior knowledge with each diagnosis. As predicted, there was no effect of diagnosis on task performance for the fictional label group. These results provide new insight on the impact of learning strategy and prior knowledge in fostering transfer of learning, potentially contributing to expert development in medicine.

## Significance statement

Diagnostic reasoning is a complex task that requires retrieving information from a variety of sources, including previously encountered patient cases as well as established prior knowledge. An essential skill for diagnostic reasoning and a core component of expertise is the ability to effectively transfer learned information from prior cases to diagnose a new case. In this report, we leveraged findings from classic cognitive psychological research to show that engaging novice diagnosticians in active retrieval when exposed to patient cases enhanced the ability to transfer of learning to novel cases. We also found that prior knowledge about diagnoses affected the ability to engage in transfer of learning, indicating that prior knowledge gained outside of a training context affects diagnostic reasoning. Together, our results provide new insights in the role of learning and knowledge on guiding the reliance on previous cases for diagnosis. These findings further our understanding of how case-based knowledge, and rehearsal strategy, can support medical learners in developing diagnostic expertise.

## Introduction

In medicine, diagnostic reasoning requires learners to gain new knowledge about diseases as well as efficiently apply that knowledge to new situations to make diagnoses, referred to as transfer of learning (Barrows & Feltovich, 1987; Boshuizen & Schmidt, 1992; Norman, 2005; Woods, 2007). Expertise in diagnostic reasoning has been characterized in a variety of different ways—from accuracy (Eva, 2005; Monteiro et al., 2019; Norman et al., 2007; Wood, 2014) to speed Sherbino et al., 2012) to adaptability (Croskerry, 2018; Mylopoulos & Woods, 2009)—and consistent across these definitions is the idea that expert diagnosticians effectively engage in transfer of learning. Thus, an important question to ask is how to effectively facilitate transfer of learning in order to promote the development of expertise in early medical learners. To answer this question, we turned to research in cognitive psychology that has demonstrated that transfer of learning is engaged when information is learned actively and through experience (for a review, see Roediger & Butler, 2011). Thus, the aim of the current study was to explore how promoting an active retrieval strategy when learning diagnostic cases (i.e., exemplars of patients that require diagnoses) affects transfer of learning in early learners.

A historical finding in cognitive psychology is that repeatedly rehearsing information during learning enhances memory for the rehearsed information, which is more likely to be used to guide subsequent decisions (Ebbinghaus, 1885). However, the way that information is rehearsed during learning—the strategy implemented—is a determining factor of the effects on memory (Craik & Lockhart, 1972). Research has noted a distinction between passive rehearsal and active retrieval learning strategies (Karpicke & Roediger, 2008; Nairne, 1986; Roediger & Butler, 2011). Whereas passive rehearsal involves repeating information during encoding (e.g., rote memorization), active retrieval involves transforming or manipulating information in the mind. An example of active retrieval is practicing recall (testing) right after encoding. The evidence suggests that engaging in active retrieval during learning creates a stronger and more flexible memory trace than engaging in passive rehearsal, often referred to as a retrieval practice or testing effect (Roediger & Butler, 2011). For example, a landmark study had participants learn a series of vignettes, either by repeatedly restudying the vignettes or by answering questions about the vignettes, therefore engaging in active retrieval. The participants were tested for their ability to recall as well as apply information they learned from the vignettes one week later. Only the vignettes that were learned via answering questions, a form of active retrieval, improved participants’ ability to recall as well as apply what they learned from the vignettes (Butler, 2010; also see, Butler et al., 2017). A recent meta-analysis revealed that, across several learning situations including medical diagnoses, engaging in active or elaborated rehearsal strategies during learning is a determining factor for how well a person can later answer concept-based and application-based questions (Pan & Rickard, 2018).

Although the benefit of active retrieval has been explained in several ways, all of these explanations share the idea that an active learning strategy effectively engages particular episodic memory processing during learning (Gureckis & Markant, 2012). According to multiple memory systems models (e.g., Ashby et al., 1998; Schacter & Tulving, 1994), different types of representations of experiences are processed in distinct modules with different properties. Within the episodic memory system, representations of past events can be formed at a general and flexible level or as a very rote “reproductive” representation. Classic memory theory suggests that learning with active retrieval, in comparison to passive rehearsal, promotes the creation of a generalized memory representation—one that captures the gist aspect of an event (Underwood, 1969; Reyan & Brainerd, 1995). This has been confirmed with more recent cognitive neuroscientific findings illustrating that forming generalized memory representations imbue mnemonic flexibility that are more easily be applied to new scenarios, supporting transfer of learning (Eichenbaum & Cohen, 2001). Moreover, engaging in active retrieval might also help promote a stronger incentive to engage in the acquired material that rote rehearsal further promoting flexibility in the use of memory, as predicted by motivation-cognitive theories (Maddox & Markman, 2010).

Transfer of learning is a central component of case-based reasoning (CBR), where one solves a current task by retrieving a similar past scenario (Kolodner, 1992). CBR is often employed in real-world reasoning scenarios that are not clearly defined (i.e., no established specific means to reach a solution), and it is also a core component of modern medical education (Eshach & Bitterman, 2003). CBR is a frequent and well-used approach in medical education as one way to gain practice recognizing and translating patient-described symptoms that are more opaque than learned lists into the clinical language of signs and symptoms (Dore et al., 2012; Lingard et al., 2003; Young et al., 2007).

Research has suggested that CBR is one way to engage in diagnostic reasoning as it flexibly draws on previous patient cases that have some similarity in symptoms or characteristics with a current case to help shape diagnostic reasoning (Eva, 2005; Norman, 2005; Young et al., 2007, 2011). Indeed, several reports have shown that expert diagnosticians increasingly rely on previous experiences to solve current problems (Dore et al., 2012; Eva, 2005; Norman, 2005; Sherbino et al., 2012), indicating that CBR as a learning tool could lead to more expert-like behaviour in diagnostic reasoning. In fact, a recent report described the efficacy of engaging in CBR for medical learners. This study found that students that learned via case-related readings and simulated patient cases (via the use of actors) showed significant improvements on a clinical assessment, due to an enhanced ability of the students to engage in transfer of learning, when compared to a control group (Turk et al., 2019; although see Himmelbauer et al. (2018) for a discussion of the importance of affect in determining the benefit of simulated cases on medical learning).

The main aim of the current study was to unite the above-described lines of research to explore how the learning strategy used in CBR (active versus passive rehearsal) impacts transfer of learning during a diagnostic reasoning task in early learners. Specifically, we tested the hypothesis that transfer of learning will be enhanced when learners engaged in active retrieval, compared to passive rehearsal, during a diagnostic task using written patient cases. To test this hypothesis, we implemented a between-subjects experimental design in which we manipulated participants’ strategy when learning about case vignettes (Experiment 1). One group of participants studied example cases by reading and then freely recalling the cases from memory (active retrieval) and another group studied these example cases via reading the cases twice in the practice session (passive rehearsal). Across the groups, we controlled key factors known to alter learning, such as the presence of feedback and exposure (Roediger & Butler, 2011). In our design, we used “real-world” diagnostic labels (e.g., Schizophrenia, Mania) that learners likely have different levels of familiarity with or pre-existing conceptual knowledge about. Following theories that suggest that established familiarity and knowledge with a concept can affect associated memory and reasoning tasks (Gilboa & Marlatte, 2017), we further explored for differences in transfer of learning across diagnoses. Following results from this exploratory analysis that indicated the presence of diagnostic label differences in transfer of learning, we conducted a second Experiment that tested if these differences across diagnostic labels would remaining without the presence of real-world labels, effectively removing access to established familiar knowledge of the diagnoses (Ashby & Maddox, 1993; Bordage & Zacks, 1984; Brooks, 1978; Brooks et al., 1991; Hatala et al., 1999; Hintzman, 1986; Medin, 1989; Medin & Schaffer, 1978; Young et al., 2007).

## Experiment 1

### Design overview

**Fig. 1:**
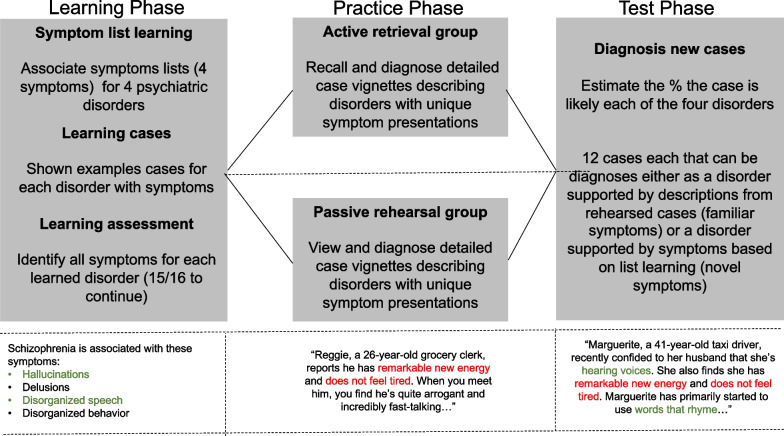
A schematic of the experimental design with an example of the presented stimuli used in each phase provided below for illustrative purposes. Used case vignettes also included diagnostically irrelevant, case-specific information (e.g., familial status, location of residence, etc.). Practice example case vignettes contained all four symptoms of the relevant diagnosis. Test case vignettes contained two familiar symptom descriptions supporting one diagnosis from the practice cases and two novel symptom descriptions supporting another diagnosis

This experiment included three phases (Fig. 1): a learning phase in which participants learned a list of symptoms associated with the four diagnoses included in the experiment; a practice phase in which one group of participants (active retrieval group) learned and recalled detailed descriptions of case vignettes and another group (passive rehearsal group) instead read these vignettes twice, gaining similar exposure to the example cases without active engagement; and a test phase in which all participants categorised new “test” case vignettes. These test vignettes were associated with two equally valid diagnoses: one diagnosis that was supported by two familiar symptom instantiations (i.e., case-specific detailed descriptions of symptoms) drawn from an earlier practice case, and one diagnosis supported by two novel symptom descriptions.

### Participants

In order to study the influence of practice approach on early learners, we invited novices (i.e., those with no formal undergraduate medical education training) to participate in this study. Sixty-four entry-level undergraduate psychology student participants were recruited from McGill University’s Psychology Human Participant Pool. All participants were fluent in English and had normal or corrected-to-normal vision. Tested participants were excluded from analysis if they withdrew (*n* = 1), had a history of major head injury, seizures, or disability (*n* = 5), had implausibly short test times (i.e., 2 *SD*s below the mean; *n* = 2), or did not complete the task as instructed (i.e., when asked to recall details from patient case vignettes, they instead inferred the diagnoses; *n* = 1). In all, 48 females and 7 males were included for analysis, with ages ranging from 18 to 34 (*M* = 20.8 years, *SD* = 2.3). The active retrieval group included 28 participants (24 female), and the passive rehearsal group included 27 participants (24 female). Informed consent was obtained from all participants.

### Stimuli

The stimuli set for the learning phase included four symptom lists associated with four common psychiatric diagnoses (mania, schizophrenia, paranoid personality disorder (PPD), and obsessive compulsive disorder (OCD); Table 1), each adapted from the diagnostic rules listed in the Diagnostic and Statistical Manual for Mental Disorders (4th ed., tex rev.; *DSM-IV-TR*; American Psychiatric Association, 2000; similar to those used in Young et al., 2007, 2011; material available upon request). There was also a written example case vignette for each diagnosis. The stimuli set for the practice phase included three written example case vignettes for each diagnosis that contained all four symptoms, presented in a unique manner (e.g., the symptom “hallucinations” would be presented as “she is following something with her eyes that no one else can see”) and contained personally identifying, specific episodic content (e.g., name, age, type of employment, familial situation). Finally, the stimuli set for the test phase included 12 test case vignettes that were designed to contain two equally probable diagnoses—each case contained novel personally identifying “patient” information, as well as two familiar symptom instantiations drawn from an earlier practice case supporting one diagnosis, and two novel symptom descriptions supporting another diagnosis. Across the 12 test cases, all four diagnoses were equally paired with every other diagnosis, and all diagnoses appeared as both the familiar and novel instantiated features.

**Table 1:** Medical diagnoses and symptoms used, adapted from Young et al., (2007, 2011)

Diagnosis (standard label)	Symptom
Mania	Decreased need for sleep
	Increased energy
	Inflated self-esteem
	More talkative
Schizophrenia	Hallucinations
	Delusions
	Disorganized speech
	Disorganized behaviour
Paranoid personality disorder (PPD)	Concerns over infidelity of spouse
	Distrustful of others
	Reads hidden meaning into daily events
	Reluctant to confide in others
Obsessive compulsive disorder (OCD)	Repetitive behaviours
	Recurring thoughts
	Difficulty ignoring thoughts
	Interferes with day-to-day life

### Procedure

All materials were presented on a computer screen, programmed with RunTime Revolution Version 2.5 (RunTime Revolution Ltd, Edinburgh, UK).

#### Learning phase

This phase involved learning the four diagnoses in an order randomly assigned to each participant. For each diagnosis, participants first studied the four associated symptoms in list form (e.g., the symptoms for mania were: increased energy, decreased sleep, inflated self-esteem, and more talkative). After learning the symptoms for a diagnosis, participants took a quiz in which they had to identify the four symptoms of the diagnosis from a list of 16 symptoms. If they did not correctly identify all four symptoms, participants re-studied the symptom list for that diagnosis and took the quiz again; this process was repeated until they passed the quiz. Participants then were shown an example case of each diagnosis which contained all four of the associated symptoms, and they identified the symptoms by typing them into text boxes. They were then shown the correct symptoms in the text boxes, and relevant text in the case vignette was highlighted. Once all four diagnoses had been studied in this manner, participants were shown the full list of 16 symptoms and had to identify the four symptoms for each diagnosis. Participants needed to correctly match 15 of the 16 symptoms to the corresponding diagnosis to advance to the practice phase. This ensured that all participants were equally familiar with the diagnoses as presented in the experiment, prior to the practice phase.

#### Practice phase

Participants were randomly assigned to either the active retrieval or passive rehearsal group. Both groups were shown 12 case vignettes that contained unique instantiations of all four symptoms learned during the learning phase, as well as other episodic details that were unrelated to any diagnosis (see Fig. 1 for an adapted example vignette). Both groups of participants first studied a case and reported their diagnoses by typing a percent likelihood (i.e., diagnostic probability) in a text box beside the name of each diagnosis. Participants were told to distribute their percentages as they saw fit, with the only restriction being that they must sum to 100%. They were also asked to report the symptoms they thought were relevant for the diagnosis in each case by typing the symptoms into text boxes. Participants were free to report either the symptom label (e.g., “more talkative”) or the symptom in its “instantiated” form (e.g., “incredibly fast-talking”) and received feedback regarding the correct diagnosis (i.e., the diagnosis that was represented in the case). Participants in the passive rehearsal group saw the example case vignette again and assigned probabilities a second time. The active retrieval group was shown a blank text box and asked to type all the details they could remember from the example case vignette. These participants were instructed that no detail was too small to remember, and they had no time limit. After recalling these details, they assigned probabilities to the four possible diagnoses for this case. This process was repeated until participants had seen and diagnosed all 12 example case vignettes, which were presented in random order.

#### Test phase

In this phase, both participant groups were presented with the same set of 12 test case vignettes in random order. As outlined in the above description of experimental stimuli, each test case included two familiar symptom descriptions that were drawn from one of the cases from the practice phase (i.e., the symptoms were described in the same form as in the cases), as well as two “novel” symptom descriptions in the context of a written case vignette. These novel symptom descriptions had not been seen by participants before and were unique instantiations of the learned symptom lists associated with each diagnosis. For each case, participants were again asked to report their diagnoses by assigning a percent likelihood to each of the four diagnoses (diagnostic probability). If participants are referencing all four symptoms equally to assign these percentages, then they should assign diagnostic probabilities as: 50% to the diagnosis supported by the familiar symptom descriptions, and 50% to the diagnosis supported by the novel symptom descriptions. However, if a participant is biased towards using information from the familiar practice cases, then there will be a deviation in the diagnostic decision from 50:50 in favour of the diagnosis supported by the familiar descriptions (aligned with Young et al., 2007, 2011). Thus, our outcome variable was the percentage assigned to each diagnosis, or diagnostic probabilities. The diagnostic probability assigned to the diagnosis supported by *familiar symptom descriptions* was our metric of transferring learning from the example cases. The order of the stimulus materials was always randomised across participants within each phase of the experiment.

### Statistical analysis

The primary analyses of interest were linear mixed models that estimated the diagnostic probabilities that participants assigned to the test cases, modelled as a function of diagnostic decision (that supported by familiar or novel symptom instantiations), diagnosis (mania, OCD, PPD, schizophrenia), experimental group (active retrieval; passive rehearsal), and the interactions between these variables, with a random intercept for participant. Independent t-tests on the average time spent in each experimental phase were conducted between the groups.

### Results

Independent t-tests on the average time spent within each phase between the groups confirmed no significant difference in the time spent during the learning phase (*t*(45) = 1.435, *p* = 0.158) nor the test phase (*t*(45) = 0.650, *p* = 0.519), yet a difference in the time spent during the practice phase (*t*(45) = 8.250, *p* < 0.001). Those in the active retrieval group (*M* = 366.6 s, SD = 1.22 s) spent longer in this phase than those in the passive rehearsal group (*M* = 148.4 s, SD = 4.30 s), which was not unexpected given the task demands of the active retrieval versus passive rehearsal experimental conditions.

**Fig. 2:**
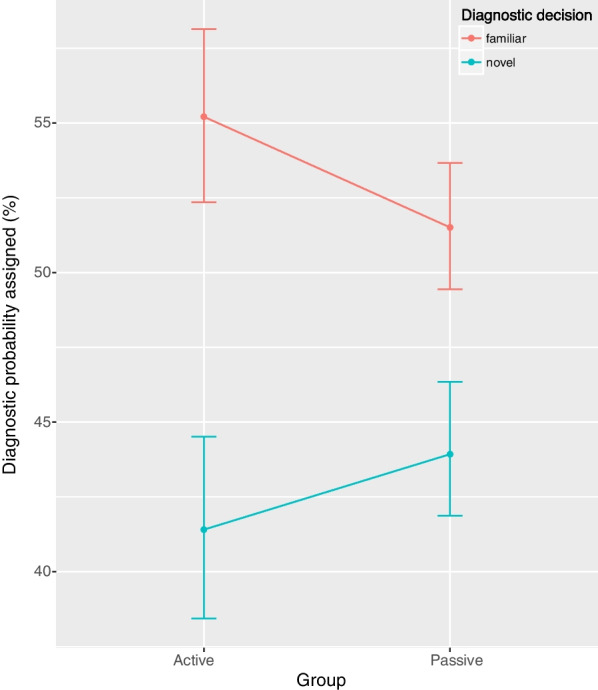
Mean diagnostic probabilities assigned (%) to the diagnosis supported by familiar symptom descriptions (reference to practice case vignettes; red) versus the diagnosis supported by novel symptom descriptions (blue) from Experiment 1. The mean diagnostic probabilities assigned to the other two of the four diagnostic options (i.e., those not intended to be represented in the case vignette) were collected but are not displayed here. Data are presented for each test group (active retrieval, passive rehearsal). Error bars represent bootstrapped 95% confidence intervals

Focusing on the results from the test phase, a linear mixed model was constructed to estimate diagnostic probability assigned to test cases with the factors of group, diagnostic decision, and diagnosis. Since practice time was different between the groups, practice time was included as a covariate in the model. This model revealed three statistically significant effects (Table 2). First, there was a main effect of diagnostic decision, such that participants, regardless of group, assigned higher diagnostic probabilities to the diagnosis supported by familiar symptom descriptions (*M* = 53.4, *SD* = 24.0) than the diagnosis supported by novel symptom descriptions (*M* = 42.7, *SD* = 24.4). Second, the factor of diagnostic decision interacted with experimental group. Compared to the passive rehearsal group, the active retrieval group assigned an even higher probability to the diagnosis supported by the familiar symptom descriptions than to the diagnosis supported by novel symptom descriptions (Fig. 2). Finally, and somewhat surprisingly, there was an interaction between diagnostic decision and diagnosis across both groups. Pairwise contrasts between levels of diagnostic decision showed that only the OCD contrast was statistically significant, *χ*^2^(1) = 8.01, *p* = 0.02, all other *p*s > 0.58, such that when OCD was the diagnosis supported by familiar symptom descriptions, participants assigned a higher probability to OCD, suggesting some role of knowledge from outside of the experimental context interacting with diagnostic labels. To explore this effect, Experiment 2 was conducted.

**Table 2:** Model parameter estimates from Experiment 1

Effect	Estimate	SE	F	*df*_*num*_	*df*_*den*_	*p*	95% CI
Group	1.16	2.41	0.23	1	52	.63	−3.56, 5.89
Diagnosis	–	–	0.33	3	1251	.80	–
Diagnostic decision	−10.69	1.30	67.25	1	1251	< .001 *	−13.25, −8.14
Group × Diagnosis	–	–	0.18	3	1251	.91	–
Group × Diagnostic decision	6.22	2.61	5.69	1	1251	.02 *	1.11, 11.33
Diagnosis × Diagnostic decision	–	–	19.79	3	1251	< .001 *	–
Group × Diagnosis × Diagnostic decision	–						
	–	1.41	3	1251	.24	–	
Practice Time	–						
	–	0.75	3	52	.39	–	

Each effect was entered as a fixed effect in a multilevel linear model, along with a random intercept for participant. Type III *F* tests with Kenward-Roger estimations for degrees of freedom are reported. An asterisk (*) denotes *p* < .05

## Experiment 2

Findings from Experiment 1 suggest that transfer of learning on a diagnostic reasoning task is enhanced when patient cases are actively retrieved compared to when they are passively rehearsed. Results from this Experiment also revealed that performance on the diagnostic reasoning task differed across the given diagnostic labels, such that familiar symptoms were more likely to contribute to an OCD diagnosis than other diagnostic labels. One possible explanation is that the diagnoses included in Experiment 1 differed in terms of how much knowledge participants had about these diseases prior to the experiment. Prior work has indicated that previous experience with a given diagnosis does influence diagnostic reasoning (Dore et al., 2012; Eva, 2005; Norman, 2005; Sherbino et al., 2012). As well, research has found familiar stimuli, those with established memory representations, are more likely to facilitate memory and reasoning than less familiar stimuli (e.g., Reder et al., 2012). Thus, we hypothesized that removing differences in familiarity or pre-existing knowledge among disorder labels should reduce any distinctions in the use of the associated disorder cases for the diagnostic reasoning. To test this hypothesis, we ran Experiment 2 in which we compared diagnostic reasoning when participants were given the established labels of diagnoses to when they were given fictional diagnoses, effectively removing the ability to access associated representations.

### Design overview

In a between-subjects design, one group (standard-label group) learned and diagnosed cases with the standard names from Experiment 1 (i.e., mania, OCD, PPD, schizophrenia) and another group (fictional-label group) learned and diagnosed cases where the names for the diagnoses were replaced with fictional ones (schizophrenia was relabelled as Ritners, PPD as Patrase, mania as Blakins, and OCD as Togastin), removing the influence of conceptual knowledge associated with real-world diagnostic labels. The symptoms and case vignettes associated with each diagnosis remained the same across groups and both groups engaged in an active retrieval learning strategy, the condition from Experiment 1 in which the effect of familiar feature descriptions was the most pronounced. All other components of the design and approach to analysis were held constant.

### Participants

Sixty-four participants were recruited; however, three participants did not complete the task, leaving 61 participants (52 female, 9 male) for analysis. Their ages ranged from 18 to 26 (*M* = 20.3 years, *SD* = 1.4). The standard-label group included 29 participants (27 female), and the fictional-label group included 32 participants (25 female).

### Results

First, independent t-tests on the average time spent revealed there was no significant difference between the two groups in terms of the time spent during the test phase (*t*(59) = 1.259, *p* = 0.213) nor the practice phase (*t*(59) = 0.420, *p* = 0.676), yet a difference in the time spent during the learning phase (*t*(59) = 4.563, *p* < 0.001). Those in the fictional label group (*M* = 1028 s, SD = 356 s) took significantly longer to learn the symptom lists than the standard label group (*M* = 679 s, SD = 217 s), suggesting learning was facilitated by the presence of ‘real’ diagnostic labels.

**Fig. 3:**
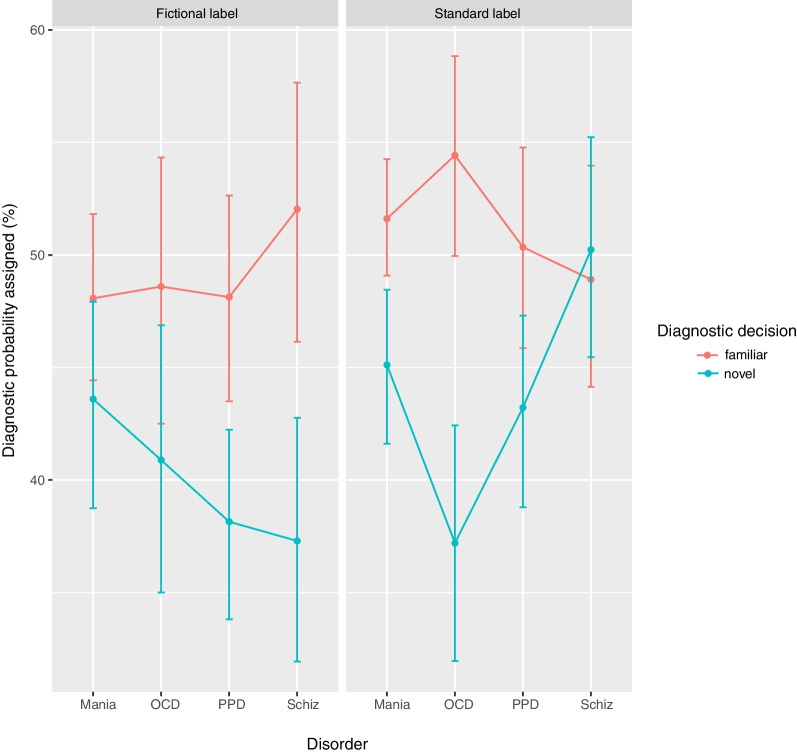
Mean diagnostic probabilities (%) assigned to the diagnosis supported by familiar symptom (red) versus the diagnosis supported by novel symptom descriptions (blue) across diagnosis (mania/Blakins, OCD/Togastin, PPD/Patrase, schizophrenia/Ritners) for the standard-label and fictional-label group, both who engaged in active retrieval in Experiment 2. Error bars represent bootstrapped 95% confidence intervals

A linear mixed model estimated diagnostic probability assigned to test cases from the factors of group (standard label, fictional label), diagnostic decision (familiar, novel), diagnosis and the interaction of these factors. We included learning time as a covariate in the model as learning time was significantly different across groups (see Table 3). Replicating Experiment 1, there was a main effect of diagnostic decision, such that participants tended to assign higher diagnostic probabilities to the diagnosis supported by familiar symptom descriptions (*M* = 50.2, *SD* = 23.8) than to the diagnosis supported by novel symptom descriptions (*M* = 41.9, *SD* = 24.0). The only other significant effect was a three-way interaction between group, diagnostic decision, and diagnosis, suggesting that the diagnostic labels affected diagnoses differently depending on whether participants learned a standard or fictional label (Fig. 3). To investigate this, we examined the two-way interaction between diagnostic decision and diagnosis separately within each experimental group. For the standard-label group (Table 4), we found a main effect of diagnostic decision: participants tended to assign higher diagnostic probabilities to the diagnosis supported by familiar symptom descriptions (*M* = 51.3, *SD* = 20.6) than to the diagnosis supported by novel symptom descriptions (*M* = 43.9, *SD* = 22.0) and replicated the interaction between diagnostic decision and diagnosis from Experiment 1. The contrast between the diagnostic probability assigned to the familiar versus novel symptom description diagnosis was statistically significant only for OCD, *χ*^2^(1) = 29.1, *p* < 0.001, all other *p*s > 0.07, also replicating findings from Experiment 1. For the fictional-label group (Table 5), while there was also a main effect of diagnostic decision as participants assigned higher probabilities to the diagnosis supported by familiar symptom descriptions (*M* = 49.2, *SD* = 26.4) than to the novel diagnoses (*M* = 40.0, *SD* = 25.6), there was no statistically significant interaction between these symptom description diagnoses and diagnosis (Fig. 3). In other words, when fictional disease labels were used, the previously documented effect of OCD receiving more diagnostic probability was no longer present.

**Table 3:** Model parameter estimates from Experiment 2

Effect	Estimate	*SE*	*F*	*df*_*num*_	*df*_*den*_	*p*	95% CI
Group	2.05	1.44	2.01	1	58	.16	−0.78, 4.89
Diagnosis	–	–	0.86	3	1389	.46	–
Diagnostic decision	−8.31	1.24	44.56	1	1389	< .001 *	−10.74, −5.87
Group × Diagnosis	–	–	0.43	3	1389	.73	–
Group × Diagnostic decision	1.84	2.49	0.55	1	1389	.46	−3.03, 6.72
Diagnosis × Diagnostic decision	–	–	1.50	3	1389	.21	–
Group × Diagnosis × Diagnostic decision	–	–	4.67	3	1389	.003 *	–
Learning time	–	–	1.79	3	1389	.19	–

Each effect was entered as a fixed effect in a multilevel linear model, along with a random intercept for participant. Type III *F* tests with Kenward-Roger estimations for degrees of freedom are reported. An asterisk (*) denotes *p* < .05

**Table 4:** Multilevel model parameter estimates in standard-label group from Experiment 2

Effect	*Estimate*	*SE*	*F*	*df*_*num*_	*df*_*den*_	*p*	95% CI
Diagnosis	–	–	1.09	3	660	.35	–
Diagnostic decision	3.69	0.80	21.38	1	660	< .001 *	2.13, 5.25
Diagnosis × Diagnostic decision	–	–	5.67	3	660	< .001 *	–

Each effect was entered as a fixed effect in a multilevel linear model, along with a random intercept for participant. Type III *F* tests with Kenward-Roger estimations for degrees of freedom are reported. An asterisk (*) denotes *p* < .05

**Table 5:** Multilevel model parameter estimates in fictional-label group from Experiment 2

Effect	*Estimate*	*SE*	*F*	*df*_*num*_	*df*_*den*_	*p*	95% CI
Diagnosis	–	–	0.35	3	729	.79	–
Diagnostic decision	4.61	0.94	24.35	1	729	< .001 *	2.79, 6.44
Diagnosis × Diagnostic decision	–	–	1.33	3	729	.26	–

Each effect was entered as a fixed effect in a multilevel linear model, along with a random intercept for participant. Type III *F* tests with Kenward-Roger estimations for degrees of freedom are reported. An asterisk (*) denotes *p* < .05

#### Online ratings

To understand why participants appeared to be differentially influenced by familiar symptom descriptions across the four diagnoses, particularly OCD, when the standard labels were given, we collected ratings about knowledge and familiarity of these diagnostic labels from an independent sample of participants. Seventy-one individuals on Amazon’s Mechanical Turk platform (*MTurk.com*), drawn from the general North American population were recruited to rate the four diagnoses. These individuals were presented with each diagnostic label (i.e., mania, OCD, paranoid personality disorder, and schizophrenia) in random order, and provided ratings of familiarity (“How familiar are you with the psychiatric diagnosis X?”, where X represents each of the four diagnosis) on a scale from 0 to 100 (0 = “I haven’t heard of this condition”; 50 = “I have heard about this condition before, but don’t know much about it”, 100 = “I am extremely knowledgeable about this condition”; McRae et al., 2005), and commonality (“How common do you think X is in relation to other psychiatric diagnosis?”) on a scale from 0 to 100 (0 = “one of the least common psychiatric diagnosis”; 50 = “about average”; 100 = “one of the most common psychiatric diagnosis”).

We ran two separate linear mixed models for ratings of familiarity and commonality as a function of diagnosis, with a random intercept for participant. The model of familiarity ratings indicated that the diagnostic labels differed in familiarity, *F*(3, 209) = 61.8, *p* < 0.001, *R*^*2*^ = 0.47. OCD was rated as the most familiar diagnosis (*M* = 69.16, *SD* = 17.01), followed by schizophrenia (*M* = 59.8, *SD* = 18.84), mania (*M* = 44.56, *SD* = 27.24), and then PPD (*M* = 29.55, *SD* = 28.16). Similarly, the model of diagnostic commonality indicated that the diagnostic labels differed, *F*(3, 209) = 28.8, *p* < 0.001, *R*^*2*^ = 0.29. OCD was rated as the most common diagnosis compared to the other diagnoses [*M* = 58.73, *SD* = 22.91; schizophrenia (*M* = 47.23, *SD* = 23.19), mania (*M* = 37.87, *SD* = 24.24), and PPD (*M* = 33.69, *SD* = 22.51)]. These ratings indicate that OCD is a highly familiar and common diagnosis. From Experiment 2, OCD was the diagnosis that showed the largest influence of familiar symptom descriptions, which could be due to this diagnostic label having a high level of familiarity or associated prior conceptual knowledge across a broad sample of participants.

## General discussion

Transfer of learning—when learning a particular set of information impacts performance on a later related task (Perkins & Salomon, 1992)—is an important component of diagnostic reasoning, as every new diagnostic case encountered can draw on previous experience. Research from cognitive psychology has suggested that actively rehearsing information during learning facilitates the formation of a dynamic and flexible form of memory that can efficiently applied to new scenarios (Eichenbaum & Cohen, 2001). The goal of the present study was to extend this research finding to the field of medical education, testing the specific hypothesis that facilitating active retrieval of diagnostic (i.e., patient) cases would enhance the use of these cases—transfer of learning—to novel cases. Across two behavioural studies, we found that novice learners (undergraduate students without knowledge of medical diagnostics) favoured using information from previous patient cases to diagnose new cases, replicating prior work (Young et al., 2007, 2011). This reliance on previous patient cases was amplified when the previous patient cases had been actively rehearsed (Experiment 1). In addition, we found differences in how participants diagnosed the four psychiatric diagnoses used in our experiments. In Experiment 2, we tested the hypothesis that differences in diagnosis were due to familiarity or prior knowledge of the diagnostic labels, which was also confirmed by a survey of online ratings of familiarity and commonality. We discuss possible interpretations and implications of these results in detail below.

Participants more readily used past patient cases to diagnose new cases if those past cases were actively rehearsed. This suggests active retrieval best supports learners to mobilize and apply information from complex cases to later diagnostic tasks. To interpret this result, we turn to cognitive research on the influence of how episodic memories are formed during learning to influence the subsequent use of these memories for reasoning tasks (Sheldon et al., 2019; Biderman et al., 2020; Delgado & Dickerson, 2012; Doll et al., 2015; Hawkins & Hastie, 1990; Madan et al., 2014). Drawing on this work, one explanation is that active retrieval helps to form a more general episodic memory representation that prioritizes interpreting the important gist-level details of the cases that are easily accessed and applied to similar yet new situations (Butler et al., 2017). In contrast, repetition may lead to forming more specific or rote-rehearsed memories that focus on highly specific details without such interpretation, rendering these memories more rigid and less likely to influence later decision making.

The above interpretation fits with classic prototype theory of memory, the ability to access a generalize representation of a category (e.g., disease) fosters flexible use of information (Jamieson et al., 2022; Rosch, 1975). There are also data to suggest that this more general form of representation is used to a greater degree by experts of a given domain (Van Overschelde et al., 2005). Specific to medicine, researchers have found that when the eye-movements of expert and novice radiologists were recorded, expert radiologists were more likely to encode and retrieve case information from a global perspective, processing the general aspects of a case rather than focusing on the idiosyncratic case-specific details, and this was associated with effective reasoning (Donovan & Litchfield, 2013; Krupinski, 1996; Kundel et al., 2007, 2008). There are other interpretations of the benefit of active retrieval for later diagnostic reasoning. One alternate interpretation is that the active retrieval group was able to represent the symptom descriptions in the patient cases verbatim and then use those to match to the symptom descriptions included in the test cases. This interpretation fits with the view that active retrieval enhances case-based reasoning, but suggests it is not due to the formation of generalized representations, but in fact specific representations. Going forward, it would be worthwhile to experimentally examine whether the general or specific detail overlap between past cases to novel cases drives transfer of learning (Brooks & Hannah, 2006; Kolodner, 1992).

In our study, we also found that diagnostic label influenced participants diagnosis. When “real” diagnostic labels were used, particularly OCD, participants assigned more diagnostic probability to the diagnosis supported by the familiar symptom descriptions. This was muted when the standard diagnostic labels were replaced with fictional (or nonsense) diagnostic labels (Experiment 2). To further contextualize this finding, we collected an online sample of familiarity and commonality ratings of the four tested diagnoses, which indicated that OCD was rated as the most familiar diagnosis. This result led us to speculate that familiarity with, or prior knowledge of, a diagnosis could be driving the reported effect, which is in accord with theories that describe how familiarity or prior knowledge, information supported by semantic memory, impacts the formation and retrieval of episodic memories (Gilboa & Marlatte, 2017). Based on these theories, we consider that the enhanced familiarity or knowledge associated with OCD led to different episodic memory formation during learning and ultimately the use of a different diagnostic strategies. This consideration raises questions about the nature and impact of familiarity with a diagnostic label. First, there is a question of the source of enhanced familiarity with diagnoses, and specifically OCD. Sources could include higher levels of media coverage known to affect the perception of a disease (Young et al., 2008), or more direct contact with individual with OCD or even enhanced familiarity with the diagnostic label itself. Second, there are questions about the nature of the reported diagnostic familiarity effect. In Experiment 2, the presence of novel diagnostic names reduced the observed differences in how the disorders were diagnosed, despite the symptoms associated with the diagnoses being identical across novel and familiar diagnostic names. This finding suggests that the disorder label is contributing in some way to how associated patient cases are represented in memory. The precise mechanisms underlying this contribution are worth further investigation and could be driven by familiar labels offering access to related experiences, interfering with or even enhancing motivation when learning about diagnostic cases (Maddox & Markham, 2010).

The findings from these studies open several avenues for future research. For example, future work could examine whether there are individual differences (e.g., age, gender, prior education) that influence the benefit of active retrieval to learning. To this point, it is worth noting that most of the participants in our in-laboratory experiments were female, due to the high proportion of female psychology students enrolled in the local university participant pool. Some evidence suggests that there are gender differences in performance on episodic memory tasks (Herlitz & Rehnman, 2008; Herlitz et al., 1997; Pauls et al., 2013); however, the gender ratio was similarly skewed towards females in all our experimental groups, making it unlikely that gender differences could explain the reported pattern of findings.

Our results also have practical implications for effectively teaching case-based reasoning (Irby, 1994; Mancinetti et al., 2019). Foremost, our results suggest that learners may benefit by engaging in active retrieval learning strategies to optimize the benefits of case-based reasoning, as active retrieval should enhance the ability of learners to engage in transfer of learning. To reach this benefit, active retrieval could be integrated throughout training programs to emphasize summarizing and reflecting on patient cases rather than reproducing the details from these cases—in essence, engaging in stage-appropriate case presentations, case summaries, or specific learning approaches such as self-explanation (Braun et al., 2017; Chamberland et al., 2015; Spafford et al., 2006). Prior to such integration of active retrieval strategies to education programs, it would be worthwhile to consider the amount of retrieval practice that brings benefit to diagnostic reasoning. It could be that making patient cases readily available through active retrieval leads to an overemphasis of these cases when making later diagnoses, potentially resulting in a failure to notice important new symptoms of a novel case. Finally, our results also suggest that when considering implementing active retrieval in the educational settings, it is important to understand the way prior knowledge—information accrued outside of an education or experimental setting—influences both diagnostic reasoning and the efficacy of pedagogical approaches to teaching diagnostic reasoning (e.g., using a spiral curriculum; Harden, 1999).

To conclude, our study provides new evidence that active retrieval during case-based reasoning is a helpful method to engage transfer of learning—a skill essential to expertise development in medicine. Our results also suggest that future work should focus on understanding the factors (e.g., prior knowledge) that influence the ability to effectively engage in this form of learning.

## Data Availability

Availability of data and material will be made upon request to the authors.
